# Observations on Measles

**Published:** 1802-07

**Authors:** 

**Affiliations:** Surgeon, of Uxbridge


### *Observations on Measles; communicated by* Mr. Edlin, *Surgeon, of Uxbridge.*

As you have expreffed a wifh in your truly valuable Journal, to be favoured by Correfpondents with an account of any epidemic difeafe that may appear in the diftricts in which they practife, I beg leave to fubmit to your notice a few Obfervations on the Meafles, which appeared in this town laft autumn, in an irregular form, and continued its deftructive career with little interruption; during the winter, many of the cafes affumed the ufual form, others that of an inflammatory fever, and feveral had every fymptom of malignant typhus.

The Meafles ufually makes its appearance at the commencement of the year, but the firft cafes that occurred here was about the latter end of September; in general, they were fo mild as to require no medical affiftance, but in the courfe of October a variety of cafes came under my obfervation, and in thefe the fever was not unufually fevere, nor the cough particularly troublefome; and confinement to the houfe, with moderate ate dilution, was all that circumftances at this time appeared to indicate. However, as November approached, and the weather became colder, a greater number of cafes occurred; the fever now put on a more inflammatory appearance, fome difficulty in the breathing was obfervable, the eyes were more red and inflamed than ufual, and the eruption affumed a more fiery rednefs; but in all thefe cafes, confinement to bed, fmall dofes of James's powder, and a blifter on the ftomach, were found to moderate the fymptoms; and I believe every cafe terminated favourably, not in my practice only, but in that of other gentlemen alfo.

Towards the middle of the month the attacks were more fudden, more violent while they lafted, and terminated in health or death very fpeedily. Some of them commenced like a common catarrh, and were hardly noticed by the parents till the dangerous fymptoms came on; while, in others, the eyes all at once appeared as red as blood; the pulfe became full, quick, and hard; the cough inceffant; a rattling noife in the throat came on, like that from obftructed phlegm; the breathing quick and laborious; fkin hot, parched, and dry, and not unfrequently a vomiting occurred, which, inftead of affording relief, greatly aggravated the difficulty of the breathing, and endangered fuffocation. If, on the firft attack, recourfe was had to the lancet, if leeches were applied to the breaft, the warm bath ufed, with blifters and an antiphlogiftic regimen, then thefe unfavourable fymptoms fubfided, and the eruption made its appearance, and frequently went through its feveral ftages as mildly as the attack had been violent; but if, on the contrary, the lancet, through the folicitation of parents, was with-held at the firft attack, or deferred but for a few hours, then the breathing uniformly became more oppreffed and interrupted, the trachea feemed loaded with obftructed phlegm, the countenance became full, bloated, and affumed a livid afpect, every vein being diftended with unoxygenated blood, and in a fhort time death relieved the unhappy patient from his complicated fufferings.

Another peculiarity in this epidemic was, that the cuticle in many children did not feparate after the difappearance of the eruption; and in feveral others that I particularly noticed, it came off in large flakes inftead of branny fcales; and the appearance of the rafh in others affumed fo ftriking a refemblance to the Scarlet Fever, that had it not have been for the violent cough and other meafly fymptoms, many fuch cafes occurring fingly might, upon a fuperficial view, have been confidered and treated as that diforder: But that this was not the cafe I remember fome well-marked inftances, particularly in a family of four children, where one was feized with every fymptom of Meafles; they put on a favourable appearance, went through their feveral ftages regularly, in a clear and fatisfactory manner, and terminated in a branny fcurf. Two others, while this was recovering, were attacked with fneezing, watery inflamed eyes, cough, difficult and oppreffed breathing, fever and thirft; and when the eruption came out, it was not diftinct, but an univerfal, red, fiery rafh, exactly refembling fcarlet fever, took place. When this declined, the fkin feparated in large flakes, the cough fubfided, and the patients did well. Soon after, the other child was taken ill; this had the ufual fymptoms, but more violent than the others; the eruption was out but one day and part of another, and then gradually difappeared.* In this cafe the fever did not decline, but about the tenth day of the difeafe the pulfe became quick and fmall, an orange coloured fur appeared on the tongue, extreme debility followed, with black fetid ftools, and other fymptoms of typhus fever. This child's fkin never feparated at all, and he was fupported with bark, wine, blifters, and other cordials; notwithftanding the cough, he in time finally recovered, although after convalefcence took place the debility for a confiderable time was very diftreffing.

This malignant form of the difeafe became more common as winter approached. The firft clear marked cafe of this kind that occurred was about the tenth of December, in a child of fix years of age; he had been ill eleven days, but as it *was only the Measles*, it was not judged neceffary to call in medical affiftance till a violent purging fupervened. I found on enquiry that he had had the Meafles; they went through their feveral ftages regularly, but the fkin, inftead of becoming branny, feparated in large flakes. I found him with a fmall fluttering pulfe beating 120 pulfations in a minute, his face bloated, and of a dark livid colour, mouth in a ftate of apthous ulceration, conftant delirium, breathing difficult and oppreffed, fkin hot, dry, and parched. In this fituation little could be expected from medicine, and the next day I heard he was dead. In another cafe, I was called in on the thirteenth day; in this the fymptoms were nearly fimilar, except that the pulfe was more regular, and the tongue moift and of a more florid hue. I applied a blifter to his breaft and another between the fhoulders, and gave him a mixture with decoction of bark and aromatic confection; and defired him to take for nourifhment a little wine

* In other cafes the rafh did not difappear on the acceffion of the fymptoms of debility, but changed its colour to a more purple hue.

wine and fago occafionally. The tenth day, his head ftill continuing unrelieved, and petechial fpots beginning to appear on every part of his body, I directed him to be fponged with vinegar and water, and muftard poultices were applied to his feet. An opiate procured him a few hours reft, and he drank pretty plentifully of wine and water acidulated with muriatic acid. On the morning of the 15th, a perfpiration broke out on his fkin, which was the forerunner of a rank, red eruption, which effaced the appearance of the petechial fpots; the fymptoms of debility, however, ftill kept increafing, and he lingered till the twentieth day, when he died. His fifter,* a child about two years of age, was taken ill with cough and fever about the middle of November, which continued till the 19th in the morning, when I faw her. Her breathing was oppreffed, fhe fneezed violently; her eyes were red, inflamed, and watery; her nofe ran; tongue white and furred; pulfe full and quick. She took a fpoon-full of a diaphoretic mixture once in four hours, and was confined to her bed. In the courfe of the night the meafles came out on her neck, face, and breaft; but by noon of the 20th, they had entirely difappeared, and a great number of diftinct fmall-pox puftules made their appearance. From this time the fever, in a great meafure, fubfided, and all the meafly fymptoms went off; the puftules maturated, and on the 7th day began to turn; on the eighth the cough and other fymptoms of meafles returned; and on the firft of December the eruption made its appearance; the pulfe was full and quick, the inflammation of the eyes and eye-lids was fo exceffive that blindnefs followed, and the breathing was extremely laborious, without the leaft appearance of expectoration. This affection of the breaft has been one of the moft diftreffing and unmanageable fymptoms, not in this cafe only, but in a great proportion of thofe which have occurred this winter. Two leeches were this day applied to her breafts, which bled copioufly, and a blifter was put on as foon as the bleeding had fubfided. Attention was paid to the ftate of her bowels, and fhe took a faline febrifuge draught every four hours. On the 2d, her pulfe was not fo full or quick, the breathing lefs oppreffed, and her cough not fo troublefome. The remedies were continued, and on the 3d the eruption began to decline, the appetite in fome meafure returned, and fymptoms of returning health were beginning to take place. Thefe favourable appearances continued

* This cafe I have given in detail from its peculiarity, and by its ferving to confirm the obfervations of Dr. Darwin, Mr. Hunter, and others, that the progrefs of one difeafe is delayed till the other has run its courfe.

nued till the 8th, when ſhe took a gentle aperient, which operated three times. The next day a coldneſs and ſhivering took place, which was ſucceeded by intenſe heat, quick low pulſe, oppreſſed breathing, and delirium; dry, parched tongue, and ſtools uncommonly fetid. The means that are uſually recommended in typhus fever were immediately had recourſe to, but without affording the leaſt relief; the parts where the bliſters had been applied became ſphacelated; veſications aroſe in various parts of her body, filled with gold-coloured water, from the ſize of a pea to that of a ſhilling, and when the fluid was diſcharged, directly became gangrenous; and farther, a little before ſhe died, a large tumour ſuddenly aroſe in the right ſide of her neck, which in a few hours increaſed to the ſize of a turkey's egg. How this tumour might have terminated had the child ſurvived, I cannot ſay; but paſt experience would lead one to ſuppoſe that ſuppuration * would have followed.

The uſual conſequences of the Meaſles, are inflamed eyes, and a diſtreſſing cough, which ſometimes terminates in Phthiſis Pulmonalis; but this winter they have proved as irregular, and aſſumed as great a variety of appearances as the complaint itſelf; the moſt remarkable of which I ſhall briefly beg leave to notice. The firſt is Apthous Fever; two well marked caſes of this kind have occurred, and immediately ſucceeded the diſappearance of the eruption; they gave way to bark, wine, and aſtringent gargles, joined to occaſional purging, but in both inſtances ſeveral weeks elapſed before the health was re-eſtabliſhed. Several caſes of dyſenteric purging have alſo come within the ſcope of my obſervation, attended with a moſt exquiſite tenderneſs of the abdomen, but they all terminated favourably by bliſtering, and the uſe of ſaline purgatives, joined with opium and ipecacuanha. In ſome children, where the ſymptoms were by no means untoward, the eruption came out as favourably as could be expected, and from the conſternation that ſeveral unfortunate caſes occaſioned, every poſſible care was taken to prevent a retroceſſion; yet, in ſpite of every endeavour, it ſometimes did occur, and was the harbinger of the moſt

---

* The inoculated Small-pox aſſumed a very unfavourable aſpect during the whole of laſt year; many children had the glands of the axilla inflame, which brought on a conſiderable degree of irritative fever, that terminated in a large abſceſs. As ſoon as this was ripe, and the matter diſcharged, the fever ſubſided and the wound healed. As far as I have noticed, it took place only in thoſe children who ſcratched the ſcab off the inoculated part of the arm; a ſore was the immediate conſequence, which, in a few days, was as large and deep as would cover a couple of horſe beans; but as ſoon as the glands of the axilla began to ſwell, then this ſore preſently filled up and healed.

moſt alarming ſymptoms, if not of inevitable deſtruction. In one caſe that came under my obſervation, the raſh ſuddenly diſappeared, after being out about ſix and thirty hours, and was immediately ſucceeded by a laborious reſpiration, threatening inſtant ſuffocation, with a total ſubſidence of the cough, which before had been very troubleſome. In this caſe bleeding, the warm bath, bliſters, and a mixture compoſed of Miſt. camph. conf. arom. et ſpt. ammon. ſucc. not only relieved theſe ſymptoms, but cauſed the raſh to appear again, and the child, after a difficult ſtruggle, recovered its health and ſtrength. In another child neither the fever nor cough declined on the diſappearance of the eruption, but terminated in a hectic fever in ſpite of every remedy that could be ſuggeſted. In many children, after the diſappearance of the inflammatory ſymptoms, an oppreſſion and difficulty in the breathing remained, which was ſometimes ſo diſtreſſing as to endanger ſuffocation, and notwithſtanding the continuance of the cough, and rattling of the phlegm, little expectoration took place; and at this period of the diſeaſe, in moſt caſes, veneſection appeared hazardous or inadmiſſible; however, ſome relief was always obtained from bliſters: but the moſt decided benefit reſulted from the uſe of a decoction of the ſeneka root, joined with opium and ipecacuanha, given in ſmall and repeated doſes. Before I tried this medicine I was extremely perplexed with ſeveral caſes that put on a moſt alarming appearance, when I was adviſed by a gentleman of this town, whoſe experience and extenſive practice give weight to his recommendation, to try the effects of the ſeneka, either in the decoction or ſyrup; and I was ſurpriſed to find, that in a very ſhort time this diſtreſſing ſymptom was conſiderably mitigated, and expectoration ſoon followed without endangering vomiting; a circumſtance I particularly wiſhed to avoid, as it manifeſtly increaſed the inflammation when preſent, and after it had ſubſided endangered its return, from the irritable ſtate of the lungs. And I believe I may aſſert with confidence, that in no one inſtance was I diſappointed in the effects of this medicine, which appeared to act like a charm; and notwithſtanding its bitterneſs, the children took it pretty well.

Such was the general appearance and termination of this epidemic, which continued its deſtructive career till the beginning of the preſent year, when it gradually ſubſided; and ſuch caſes as have ſince occurred have neither excited any alarm, nor been attended with any ſymptom, that could give uneaſineſs either to the parent or practitioner.

